# Genomic Analysis Identifies New Loci Associated With Motor Complications in Parkinson's Disease

**DOI:** 10.3389/fneur.2020.00570

**Published:** 2020-07-07

**Authors:** Ho-Sung Ryu, Kye Won Park, Nari Choi, Jinhee Kim, Young-Min Park, Sungyang Jo, Mi-Jung Kim, Young Jin Kim, Juyeon Kim, Kiju Kim, Seong-Beom Koh, Sun Ju Chung

**Affiliations:** ^1^Department of Neurology, Kyungpook National University Hospital, Daegu, South Korea; ^2^Department of Neurology, Asan Medical Center, University of Ulsan College of Medicine, Seoul, South Korea; ^3^Department of Neurology & Parkinson's Disease Center, Guro Hospital, Korea University, Seoul, South Korea; ^4^Department of Neurology, Dobong Hospital, Seoul, South Korea; ^5^Department of Neurology, Bobath Memorial Hospital, Seongnam-si, South Korea; ^6^Department of Neurology, Best Heals Hospital, Ansan-si, South Korea; ^7^Department of Neurology, Metro Hospital, Anyang, South Korea; ^8^Department of Neurology, The Good Light Hospital, Gwangju, South Korea

**Keywords:** genome-wide association study, genomic variants, Parkinson's disease, motor fluctuations, levodopa-induced dyskinesia

## Abstract

**Background:** Parkinson's disease (PD) is a common neurodegenerative disorder, characterized by a clinical symptomatology involving both motor and non-motor symptoms. Motor complications associated with long-term dopaminergic treatment include motor fluctuations and levodopa-induced dyskinesia (LID), which may have a major impact on the quality of life. The clinical features and onset time of motor complications in the disease course are heterogeneous, and the etiology remains unknown.

**Objective:** We aimed to identify genomic variants associated with the development of motor fluctuations and LID at 5 years after the onset of PD.

**Methods:** Genomic data were obtained using Affymetrix Axiom KORV1.1 array, including an imputation genome-wide association study (GWAS) grid and other GWAS loci; functional variants of the non-synonymous exome; pharmacogenetic variants; variants in genes involved in absorption, distribution, metabolism, and excretion of drugs; and expression quantitative trait loci in 741 patients with PD.

**Results:** *FAM129B* single-nucleotide polymorphism (SNP) rs10760490 was nominally associated with the occurrence of motor fluctuations at 5 years after the onset of PD [odds ratio (OR) = 2.9, 95% confidence interval (CI) = 1.8–4.8, *P* = 6.5 × 10^−6^]. *GALNT14* SNP rs144125291 was significantly associated with the occurrence of LID (OR = 5.5, 95% CI = 2.9–10.3, *P* = 7.88 × 10^−9^) and was still significant after Bonferroni correction. Several other genetic variants were associated with the occurrence of motor fluctuations or LID, but the associations were not significant after Bonferroni correction.

**Conclusion:** This study identified new loci associated with the occurrence of motor fluctuations and LID at 5 years after the onset of PD. However, further studies are needed to confirm our findings.

## Introduction

Parkinson's disease (PD) is a chronic, progressive neurodegenerative disorder characterized by a heterogeneous clinical symptomatology involving both motor and non-motor symptoms (1–3). The pathological hallmarks of PD are abnormal accumulation of alpha-synuclein (α-syn) aggregates, Lewy bodies, and Lewy neurites (4, 5). The α-synucleinopathy in PD involves not only dopaminergic neurons in the substantia nigra pars compacta of the midbrain but also other vulnerable neurotransmitter systems in the central nervous system (6, 7).

Levodopa is the most effective and potent medication for the treatment of motor symptoms of PD (8), and early treatment with levodopa increases life expectancy (9). However, long-term treatment of patients with PD with levodopa can result in the occurrence of motor fluctuations and dyskinesias. These late motor complications can become major causes of disability and reduce the quality of life of patients (10). To date, the pathophysiological mechanisms underlying motor fluctuations and levodopa-induced dyskinesia (LID) in patients with PD remain unclear.

Over the last two decades, rare variants of more than 20 genes have been reported to cause genetic PD (11). The common genetic risk factors for sporadic PD have been identified by genome-wide association studies (GWAS). To date, 90 independent genetic variants have been identified as risk factors for sporadic PD (12). Although previous GWAS and other genetic studies have indicated the importance of genetic contribution to the development of PD, the contribution of genetic factors to specific phenotypes of PD has not been well-studied. Identification of genetic risk factors for the major clinical phenotypes of PD may provide important insights into the underlying molecular mechanisms and valuable information for potential adjustments to overcome genetic heterogeneity in clinical trials. This GWAS aimed to identify the genetic variants associated with the occurrence of motor fluctuations and LID in patients with sporadic PD.

## Materials and Methods

### Patients

We included 741 patients who were diagnosed with PD (Supplementary Figure 1). Experienced movement disorder specialists (SJC, HSR, MJK, JK, and YJK) made the diagnosis of PD using the clinical diagnostic criteria of the United Kingdom Parkinson's Disease Society Brain Bank (13). All patients were enrolled from the clinical practice of the Department of Neurology of the Asan Medical Center, Seoul, South Korea, between January 1, 2011 and April 30, 2016. All patients were born and resided in South Korea. All patients were unrelated and ethnic Koreans without any foreign ancestry. The Institutional Review Board (IRB) of Asan Medical Center approved the study, and all patients provided an informed consent in accordance with the IRB regulations.

### Clinical Assessment

Motor fluctuations were defined as alternating between periods of good motor symptom control (on-time) and periods of reduced motor symptom control (off-time), which were dependent on the scheduled intake time of levodopa and other dopaminergic medications (14). The time between the onset of PD motor symptoms and the occurrence of motor fluctuations was assessed in each patient.

LID was defined as involuntary choreiform or dystonic body movements, which occur most frequently when levodopa concentrations are at its highest (peak-dose dyskinesia) or, less commonly, at the beginning or end of levodopa administration, or both (diphasic dyskinesia) (14). The time between the onset of PD motor symptoms and the occurrence of LID was assessed in each patient. PD onset was defined as the onset of first motor symptoms in patients with PD.

The presence of motor fluctuations or LID was determined using the clinical history and Unified Parkinson's Disease Rating Scale (UPDRS) part IV. Dystonia that occurred in the morning before taking a medication was not considered as LID (15).

### Genomic Analysis

Genotype data were obtained using the Korean Chip (K-CHIP), obtained from the K-CHIP consortium. K-CHIP was designed by the Center for Genome Science, Korea National Institute of Health, Korea (4845-301, 3000-3031) (www.cdc.go.kr). K-CHIP uses Affymetrix Axiom Customized Biobank Genotyping Arrays (Affymetrix, Santa Clara, CA, USA) and contains 827,783 variants. K-CHIP consists of an imputation GWAS grid [505,000 Asian-based grid with minor allele frequency (MAF) >5% in Asians]; exome contents [84,000 Korean-based grid with MAF >5%, in Koreans; 149,000 coding single-nucleotide polymorphisms (cSNPs); and insertions and deletions on the basis of data from 2000 whole exome sequences and 400 whole genome sequences with MAF> 0.1%]; new exome/loss of function contents (44,000 variants); expression quantitative trait loci (17,000 variants); absorption, distribution, metabolism, and excretion genes; and other miscellaneous variants.

### Sample Quality Controls

The primary sample quality control was as follows: samples with low call rate (<0.95%) were excluded from the analysis because of the possibility of low DNA quality or experimental error; high heterozygosity was excluded from the analysis because of low DNA quality or possible contamination of samples. The entire sample distribution was checked, and low-quality samples were excluded if they deviated significantly from the entire sample distribution. SNP pruning was also performed. Because cryptic first-degree relative and multidimensional scaling (MDS) analyses are very time consuming when using whole data, only representative SNP information based on linkage disequilibrium were selected from the data. Due to the possibility of population stratification, samples that deviated from the whole sample were excluded from the analysis by assessing the MDS. If there were more than a certain number of SNPs with only one sample, the possibility of errors due to DNA quality and technical artifacts was excluded.

Secondary sample quality control consisted of genotype calling, excluding samples deemed to be of low quality based on the primary sample quality control criteria and sex-inconsistent samples. Samples that did not satisfy the quality control criteria after a repeat sample quality control were excluded. SNP data were excluded from the cryptic first-degree relative analysis because statistical analysis assumes the independence for each sample in most cases.

### SNP Quality Controls

An SNPolisher analysis was performed to exclude low-quality SNPs. SNPs with low call rates were excluded when the call rate was <95% because errors in the calling process can occur due to probe design and clustering analysis problems. If the Hardy–Weinberg equilibrium (HWE) test *P*-value of a specific SNP is low, it indicates a probable error in the genotype clustering process; therefore, the HWE *P* < 10^−6^. If the frequency of a genetic variation is extremely different from that in Korean and Asian populations, there may be a genotype clustering error. Therefore, we excluded cases where the difference in MAF was >0.2. Both cases and controls were excluded if the MAF was <1%.

### Statistical Analysis

The associations of each genetic variant with the occurrence of motor fluctuations and LID were investigated using multiple logistic regression models. We used the Cochran–Armitage trend test and the Jonckheere–Terpstra test, and adjusted all analyses by sex and age at onset of PD. For each genetic variant, we calculated the odds ratio (OR), 95% confidence interval (CI), and two-tailed *P*-value. For sensitivity analyses, similar analyses were performed for patients aged ≥50 years at onset of PD to further adjust for the effects of age at onset of PD on the occurrence of motor fluctuations and LID. The *P*-values from the primary analyses were assessed for significance using the Bonferroni correction for multiple comparisons. Clustering quality control was performed by visual inspection of analytic data of SNPs with a *P* < 0.0001. Markers that did not clearly separate between different genotypes and were not closely located in the same genotype were excluded (Supplementary Figure 2). Manhattan plots and quantile–quantile plots were constructed for *P*-values for all genotyped variants that passed quality controls.

The statistical analysis was performed using the PLINK program (version 1.90, NIH-NIDDK Laboratory of Biological Modeling, Bethesda, MD, USA), Haploview (version 4.2, Daly Lab at the Broad Institute, Cambridge, MA, USA), LocusZoom (version 1.4, University of Michigan, Department of Biostatistics, Center for Statistical Genetics, Ann Arbor, MI, USA), and R (version 3.1.2, Free Software Foundation, Inc., Boston, MA, USA).

## Results

### Patients

Clinical and genotyping data were obtained from 741 patients with PD who were followed for at least 5 years after the onset of PD. The demographic and clinical features of study patients are summarized in Table 1. The study group consisted of 325 men (43.9%) and 416 women (56.1%). The mean age at onset of PD was 57.1 years, while the mean disease duration from the onset of PD to the last follow-up was 10.8 ± 4.5 years.

**Table 1:** Demographic and clinical characteristics of patients.

**Characteristic**	**Patients**
Total sample, *n*	741
Men, *n* (%)	325 (43.9)
Women, *n* (%)	416 (56.1)
Age at onset of PD, years, mean ± SD (range)	57.1 ± 0.1 (28–87)
Disease duration, years, mean ± SD (range)	10.8 ± 4.5 (5–31)
Patients with motor fluctuations, *n* (%)	554 (74.8)
Duration between PD onset and development of motor fluctuations, years, mean ± SD (range)	6.9 ± 3.4 (1–24)
Patients with levodopa-induced dyskinesia, *n* (%)	496 (66.8)
Duration between PD onset and development of levodopa-induced dyskinesia, years, mean ± SD (range)	7.2 ± 3.4 (1–21)
Patients with motor fluctuations at 5 years after PD onset, *n* (%)	219 (29.6)
Duration between PD onset and development of motor fluctuations, years, mean ± SD (range)	3.9 ± 1.1 (1–5)
Patients with levodopa-induced dyskinesia at 5 years after PD onset, *n* (%)	172 (23.2)
Duration between PD onset and development of levodopa-induced dyskinesia, years, mean ± SD (range)	3.9 ± 1.1 (1–5)
MMSE score (range)	26.1 ± 3.2 (10–30)
MoCA score (range)	22.6 ± 5.6 (3–30)

*PD, Parkinson's disease; SD, standard deviation; MMSE, Mini-Mental State Examination; MoCA, Montreal Cognitive Assessment*.

### Motor Fluctuations

**Figure 1:**
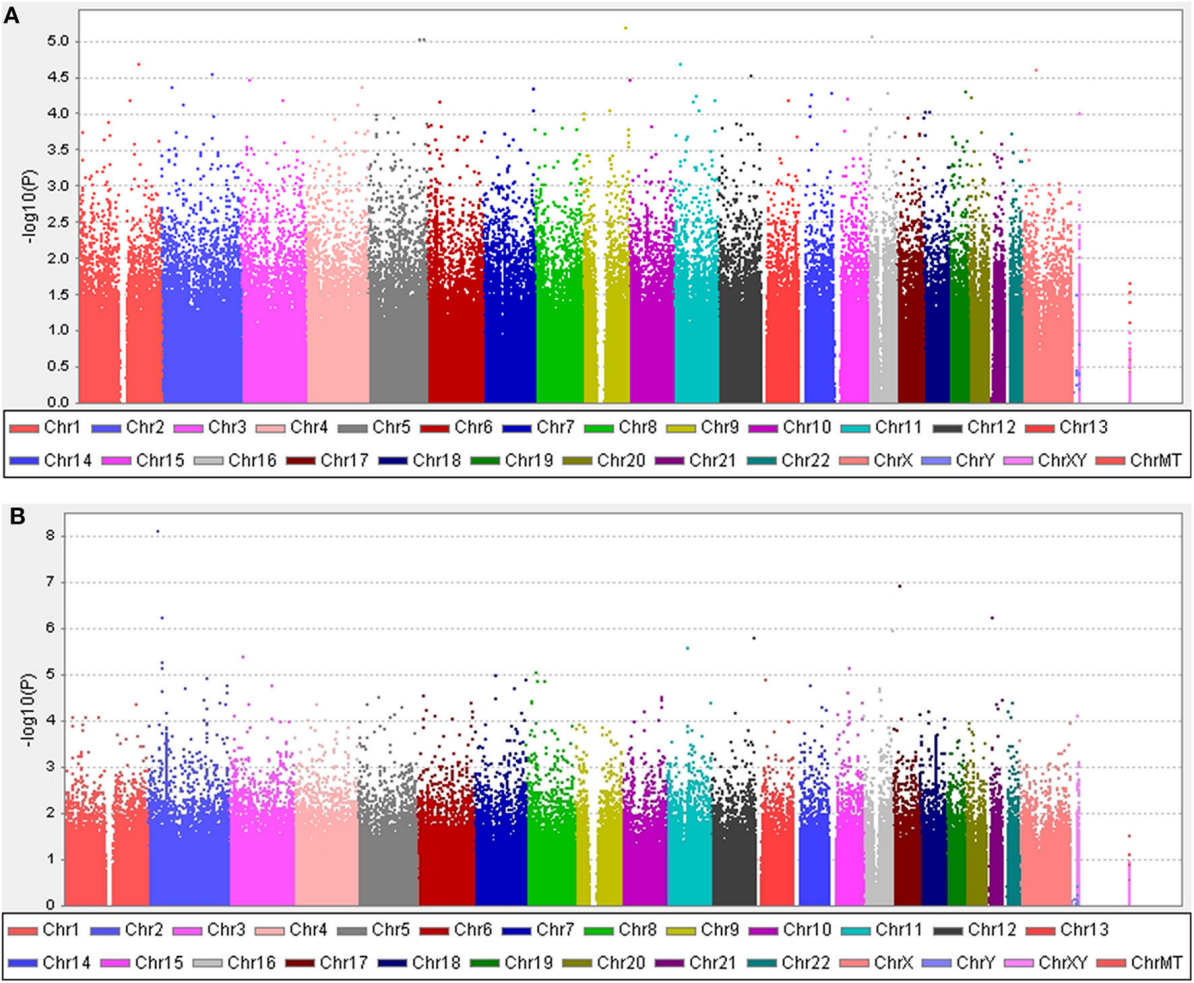
Manhattan plots. **(A)** The plot shows *P*-values for association analyses between 583,535 SNPs and the occurrence of motor fluctuations 5 years after the onset of Parkinson's disease (PD). The most significant association observed is with a locus on chromosome 9 (rs10760490). **(B)** The plot shows *P*-values for association analyses between 583,535 SNPs and the occurrence of levodopa-induced dyskinesia 5 years after the onset of PD. The most significant association observed is with a locus on chromosome 2 (rs144125291). *P*-values are log-transformed (*y*-axis) and plotted against chromosomal position (*x*-axis).

Five years after the onset of PD, 219 (29.6%) patients exhibited motor fluctuations. No difference was observed between patients with PD with motor fluctuations (92 men, 42.0%) and those without motor fluctuations (233 men, 44.6%) (*P* = 0.480) in terms of sex. The mean age at onset of PD was lower in patients with motor fluctuations than in those without motor fluctuations (54.0 ± 10.3 years vs. 58.4 ± 9.7 years, *P* < 0.001). The mean disease duration between the onset of PD and the last follow-up was shorter in patients with motor fluctuations than in those without motor fluctuations (9.5 ± 4.0 years vs. 11.3 ± 4.6 years, *P* < 0.001; Supplementary Table 1). The 583,535 SNPs that passed quality controls were genotyped and analyzed. Quantile–quantile plots were made for the presence of LID at 5 years after onset of PD (Supplementary Figure 3A), and a Manhattan plot is described in Figure 1A. The top 20 SNPs associated with the occurrence of motor fluctuations are listed in Table 2. *FAM129B* SNP rs10760490 was nominally associated with the occurrence of motor fluctuations at 5 years after onset of PD (OR = 2.9, 95% CI = 1.8–4.8, *P* = 6.5 × 10^−6^). However, *FAM129B* SNP rs10760490 and other SNPs were not significant after Bonferroni correction (Table 2).

**Table 2:** Top 20 genomic variants associated with the occurrence of motor fluctuations, in decreasing order of statistical significance.

**Gene**	**SNP**	**Chr**	**Position**	**Region relative to gene**	**Allele (minor/major)**	**Minor allele frequency (case/control)**	**OR (95% CI)**	***P*-value**
*FAM129B*	rs10760490	9	130335418	Intron	A/G	0.08/0.03	2.93 (1.80, 4.77)	6.50E−06
*SNX29*	rs150380018	16	12569788	Intron	G/T	0.04/0.01	6.53 (2.54, 16.79)	8.35E−06
*C5orf52*	rs10051838	5	157102159	Missense	A/G	0.17/0.09	2.09 (1.50, 2.91)	9.07E−06
*STK10*	rs77462941	5	171598434	Intron	C/T	0.13/0.23	0.50 (0.36, 0.68)	9.41E−06
*FAM163A*	rs6680679	1	179753147	Intron	G/A	0.14/0.07	2.15 (1.50, 3.07)	2.01E−05
*NAV2*	rs7949975	11	19985339	Intron	G/C	0.33/0.23	1.71 (1.33, 2.19)	2.05E−05
*LOC392452*	rs2022502	23	45540415	Upstream, downstream	C/T	0.20/0.10	2.39 (1.58, 3.62)	2.48E−05
*GALNT13*	rs6710932	2	154872606	Intron	A/G	0.08/0.16	0.45 (0.31, 0.66)	2.81E−05
*NFYB*	rs75845252	12	104539534	Upstream	T/C	0.09/0.04	2.62 (1.64, 4.19)	2.97E−05
*RBMS3-AS3*	rs13068014	3	29170975	Downstream	A/C	0.30/0.20	1.71 (1.33, 2.20)	3.31E−05
*AKR1C4*	rs191812506	10	5272947	Downstream, upstream	C/T	0.05/0.01	4.14 (2.01, 8.55)	3.31E−05
*GALNTL6*	rs77688865	4	172563203	Upstream, downstream	G/T	0.05/0.01	3.69 (1.90, 7.19)	4.17E−05
*GALNT14*	rs144125291	2	31106055	Downstream, upstream	T/C	0.05/0.02	3.47 (1.84, 6.52)	4.35E−05
*DPP6*	rs59309371	7	153938863	Intron	T/C	0.25/0.36	0.59 (0.46, 0.76)	4.51E−05
*CTU1*	rs117770234	19	51614232	Upstream	A/G	0.04/0.01	4.43 (2.03, 9.68)	4.78E−05
*CDH8*	rs138852987	16	61482087	Downstream, upstream	C/T	0.06/0.02	3.29 (1.79, 6.05)	5.01E−05
*DIO3*	rs11624718	14	102069522	Downstream, upstream	G/A	0.39/0.50	0.63 (0.50, 0.79)	5.20E−05
*SLC25A21*	rs8010937	14	37324893	Intron	A/C	0.13/0.07	2.12 (1.46, 3.07)	5.32E−05
*PPP6R3*	rs61188641	11	68336714	Intron	G/A	0.04/0.01	4.63 (2.05, 10.47)	5.58E−05
*LOC339593*	rs6040792	20	11597971	Upstream, downstream	C/T	0.25/0.16	1.74 (1.33, 2.28)	5.83E−05

*SNP, single-nucleotide polymorphism; Chr, chromosome; OR, odds ratio; CI, confidence interval*.

### Levodopa-Induced Dyskinesia

**Figure 2:**
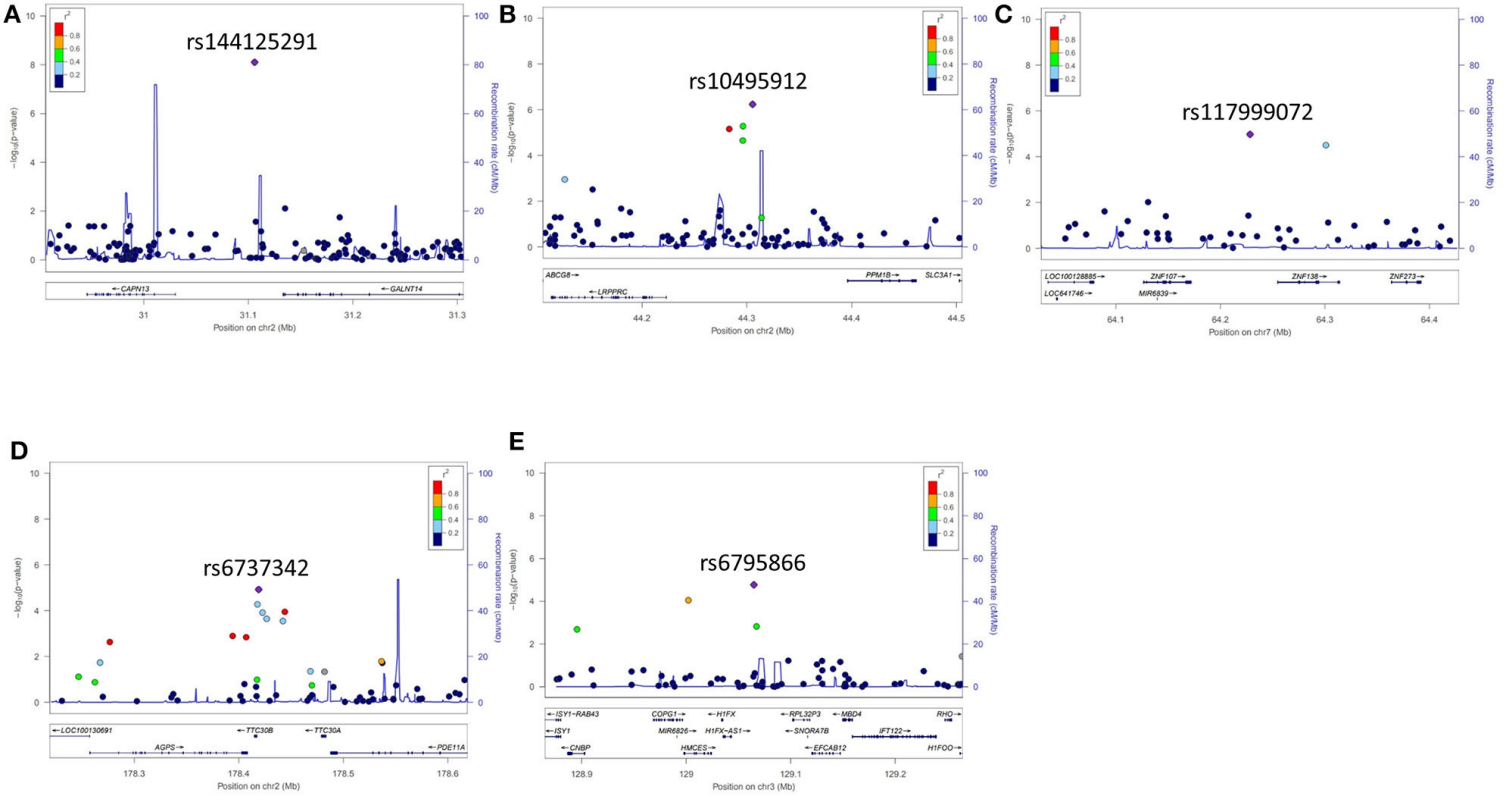
Representative regional plots for genetic variants that showed associations with the occurrence of levodopa-induced dyskinesia 5 years after the onset of Parkinson's disease. **(A)** rs144125291, **(B)** rs10495912, **(C)** rs117999072, **(D)** rs6737342, and **(E)** rs6795866.

Five years after the onset of PD, 172 patients had LID (23.2%). No difference was observed between patients with LID (75 men, 43.6%) and those without LID (250 men, 43.9%) (*P* = 0.892) in terms of sex. The mean age at onset of PD was lower in patients with LID than in those without (55.2 ± 10.7 years vs. 57.7 ± 9.8 years, *P* = 0.007). The mean duration between disease onset and the last follow-up was shorter in patients with LID than in those without LID (9.1 ± 3.5 years vs. 11.3 ± 4.6 years, *P* < 0.001; Supplementary Table 2). After quality controls, 583,379 SNPs were genotyped and analyzed. Quantile–quantile plots were made for the occurrence of LID (Supplementary Figure 3B), and a Manhattan plot is described in Figure 1B. The top 20 SNPs associated with the occurrence of LID 5 years after the onset of PD are listed in Table 3. The *GALNT14* SNP rs144125291 had the lowest *P*-value and was significantly associated with LID even after Bonferroni correction (OR = 5.5, 95% CI = 2.9–10.3, *P* = 7.88 × 10^−9^; Table 3). The representative regional association plots of rs10495912, rs117999072, rs6737342, and rs6795866 showed other risk variants within 150 kb (Figures 2A–E).

**Table 3:** Top 20 genomic variants associated with the occurrence of levodopa-induced dyskinesia, in decreasing order of statistical significance.

**Gene**	**SNP**	**Chr**	**Position**	**Region relative to gene**	**Allele (minor/major)**	**Minor allele frequency (case/control)**	**OR (95% CI)**	***P*-value**
*GALNT14*	rs144125291	2	31106055	Downstream, upstream	T/C	0.07/0.01	5.45 (2.87, 10.33)	7.88E−09
*C17orf51*	rs139221627	17	21715699	Upstream	T/C	0.07/0.02	4.68 (2.51, 8.73)	1.20E−07
*C21orf37*	rs208892	21	18813490	Intron	A/G	0.40/0.26	1.90 (1.47, 2.45)	5.74E−07
*LRPPRC*	rs10495912	2	44305461	Upstream, downstream	A/G	0.07/0.02	4.03 (2.24, 7.24)	5.81E−07
*CBFA2T3*	rs150854091	16	89028784	Intron	A/G	0.08/0.02	3.64 (2.10, 6.33)	1.12E−06
*TMEM132C*	rs1531246	12	128999121	Intron	G/C	0.18/0.09	2.28 (1.62, 3.21)	1.60E−06
*SCGB1D4*	rs953169	11	62083542	Upstream	G/A	0.45/0.31	1.80 (1.41, 2.31)	2.57E−06
*TMEM158*	rs118109628	3	45279523	Downstream, upstream	A/G	0.03/0.003	9.35 (2.96, 29.55)	3.91E−06
*LRPPRC*	rs12185607	2	44296280	Upstream, downstream	T/G	0.10/0.04	2.86 (1.79, 4.57)	5.25E−06
*ADAM10*	rs118049686	15	58895720	Intron	A/G	0.06/0.02	4.07 (2.11, 7.86)	6.93E−06
*LRPPRC*	rs17031893	2	44283172	Upstream, downstream	G/A	0.08/0.03	3.24 (1.89, 5.55)	6.96E−06
*EXTL3*	rs73564758	8	28521861	Intron, downstream	G/A	0.05/0.01	4.58 (2.20, 9.52)	8.96E−06
*ZNF138*	rs117999072	7	64228326	Upstream, downstream	A/G	0.08/0.03	3.15 (1.84, 5.37)	1.06E−05
*TTC30B*	rs6737342	2	178419117	Upstream, downstream	G/A	0.07/0.02	3.37 (1.90, 5.98)	1.19E−05
*HSPH1*	rs143639498	13	31696395	Downstream	C/T	0.05/0.01	4.48 (2.15, 9.32)	1.25E−05
*LOC389602*	rs10281583	7	155811374	Downstream	A/G	0.18/0.09	2.11 (1.50, 2.97)	1.30E−05
*DUSP26*	rs147270897	8	34132814	Intron, upstream	C/T	0.04/0.01	5.39 (2.31, 12.57)	1.36E−05
*SOX17*	rs183607239	8	55390249	Downstream, upstream	G/A	0.03/0.004	7.50 (2.59, 21.75)	1.40E−05
*RPL32P3*	rs6795866	3	129064722	Exon, downstream	G/A	0.05/0.01	4.59 (2.15, 9.80)	1.70E−05
*TMX1*	rs10129471	14	51782967	Intron, downstream, upstream	T/C	0.08/0.03	3.12 (1.81, 5.38)	1.71E−05

*SNP, single-nucleotide polymorphism; Chr, chromosome; OR, odds ratio; CI, confidence interval*.

### Sensitivity Analysis for Patients With PD Aged ≥50 Years at the Onset of PD

**Figure 3:**
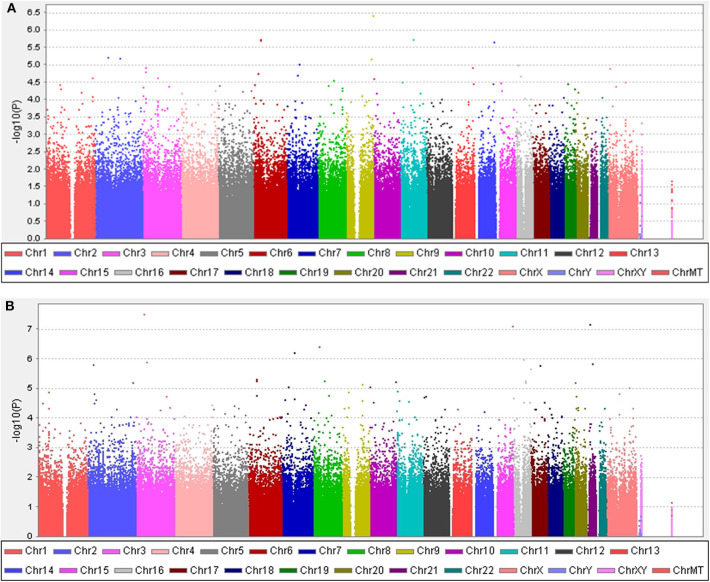
Manhattan plots. **(A)** The plot shows the *P*-values for association analyses between 580,128 SNPs and the occurrence of motor fluctuations 5 years after Parkinson's disease (PD) onset in patients aged ≥50 years at disease onset. The most significant association observed is with a locus on chromosome 9 (rs191519045). **(B)** The plot shows *P*-values for association analyses between 579,399 SNPs and the occurrence of levodopa-induced dyskinesia 5 years after onset of PD in patients aged ≥50 years at disease onset. The most significant association observed is with a locus on chromosome 3 (rs118109628). *P*-values are log-transformed (*y*-axis) and plotted against chromosomal position (*x*-axis).

The clinical features of patients with PD are presented in Table 4 and Supplementary Tables 3, 4. Five years after the onset of PD, 141 (24.4%) of 578 patients with PD exhibited motor fluctuations. A Manhattan plot is described in Figure 3A. The top 20 SNPs associated with the occurrence of motor fluctuations are listed in Table 5. *RABL6* SNP rs191519045 had the lowest *P*-value, but none of the SNPs were significant after Bonferroni correction. Representative regional association plots of rs72850586, rs76767606, and rs12408511 showed other risk variants within 150 kb (Supplementary Figures 4A–C).

**Table 4:** Demographic and clinical characteristics of patients aged ≥50 years at onset of Parkinson's disease.

**Characteristic**	**Patients**
Total sample, *n*	578
Men, *n* (%)	247 (42.7)
Women, *n* (%)	331 (57.3)
Age at onset of PD, years, mean ± SD (range)	61.1 ± 7.1 (50–87)
Disease duration, years, mean ± SD (range)	10.3 ± 3.9 (5–27)
Patients with motor fluctuations, *n* (%)	403 (69.7)
Duration between PD onset and development of motor fluctuations, years, mean ± SD (range)	7.1 ± 3.2 (1–20)
Patients with levodopa-induced dyskinesia, *n* (%)	354 (61.1)
Duration between PD onset and development of levodopa-induced dyskinesia, years, mean ± SD (range)	7.3 ± 3.2 (1–19)
Patients with motor fluctuations 5 years after PD onset, *n* (%)	141 (24.4)
Duration between PD onset and development of motor fluctuations, years, mean ± SD (range)	3.9 ± 1.2 (1–5)
Patients with levodopa-induced dyskinesia 5 years after PD onset, *n* (%)	115 (19.9)
Duration between PD onset and development of levodopa-induced dyskinesia, years, mean ± SD (range)	4.0 ± 1.1 (1–5)
MMSE score (range)	25.8 ± 3.2 (10–30)
MoCA score (range)	21.8 ± 5.7 (3–30)

*PD, Parkinson's disease; SD, standard deviation; MMSE, Mini-Mental State Examination; MoCA, Montreal Cognitive Assessment*.

**Table 5:** Top 20 genomic variants associated with the occurrence of motor fluctuations in patients aged ≥50 years at onset of Parkinson's disease.

**Gene**	**SNP**	**Chr**	**Position**	**Region relative to gene**	**Allele (minor/major)**	**Minor allele frequency (case/control)**	**OR (95% CI)**	***P*-value**
*RABL6*	rs191519045	9	139707344	Intron, exon	G/A	0.04/0.002	17.66 (3.89, 80.15)	3.81E−07
*PPP6R3*	rs61188641	11	68336714	Intron	G/A	0.05/0.01	8.40 (2.97, 23.79)	1.91E−06
*SAYSD1*	rs72850586	6	39153495	Upstream, downstream	G/A	0.05/0.01	8.40 (2.97, 23.77)	1.92E−06
*SAYSD1*	rs72850539	6	39137869	Upstream, downstream	A/G	0.05/0.01	8.36 (2.95, 23.66)	2.05E−06
*DIO3*	rs11624718	14	102069522	Downstream, upstream	G/A	0.35/0.49	0.51 (0.39, 0.68)	2.28E−06
*ANTXR1*	rs56216132	2	69377298	Intron	A/C	0.50/0.35	1.87 (1.42, 2.46)	6.07E−06
*MAP3K2*	rs147429309	2	128136163	Intron, upstream	T/G	0.05/0.01	6.99 (2.63, 18.57)	6.61E−06
*FAM129B*	rs10760490	9	130335418	Intron	A/G	0.10/0.03	3.24 (1.89, 5.55)	7.10E−06
*ZNF92*	rs190170956	7	65066217	Upstream, downstream	C/T	0.04/0.003	10.62 (2.90, 38.88)	9.74E−06
*SNX29*	rs150380018	16	12569788	Intron	G/T	0.04/0.004	8.75 (2.76, 27.69)	1.00E−05
*TBC1D5*	rs73817453	3	18117165	Intron	A/G	0.05/0.01	5.38 (2.33, 12.42)	1.20E−05
*LINC00460*	rs117816291	13	106915837	Upstream, downstream	A/G	0.05/0.01	5.38 (2.33, 12.42)	1.20E−05
*GRPR*	rs12009947	23	16108832	Upstream, downstream	T/C	0.22/0.41	0.41 (0.27, 0.62)	1.31E−05
*TBC1D5*	rs76767606	3	18064472	Intron	A/G	0.05/0.01	5.63 (2.34, 13.56)	1.61E−05
*CMAHP*	rs6456661	6	25214720	Upstream, downstream	A/G	0.05/0.01	6.04 (2.38, 15.29)	1.79E−05
*NUPR1L*	rs146088024	7	56232344	Downstream, upstream	C/A	0.06/0.01	4.98 (2.21, 11.21)	1.99E−05
*XPO6*	rs142186210	16	28138044	Intron, exon	G/A	0.05/0.01	5.96 (2.35, 15.09)	2.12E−05
*GBE1*	rs6798680	3	81905441	Intron, upstream, downstream	A/C	0.38/0.48	0.56 (0.42, 0.73)	2.40E−05
*RYR2*	rs12408511	1	237842915	Intron	T/A	0.10/0.04	2.92 (1.74, 4.91)	2.47E−05
*PFKP*	rs117516530	10	2966617	Upstream, downstream	G/A	0.17/0.08	2.31 (1.55, 3.43)	2.58E−05

*SNP, single-nucleotide polymorphism; Chr, chromosome; OR, odds ratio; CI, confidence interval*.

Five years after the onset of PD, 115 (19.9%) of 578 patients with PD had LID. A Manhattan plot is described in Figure 3B. The 20 SNPs associated with the occurrence of LID are listed in Table 6. None of these SNPs were significant after Bonferroni correction. Regional association plots of rs117999072, rs149201992, and rs6907129 showed other risk variants within 150 kb (Supplementary Figures 4D–F).

**Table 6:** Top 20 genomic variants associated with the occurrence of levodopa-induced dyskinesia in patients aged ≥50 years at the onset of Parkinson's disease.

**Gene**	**SNP**	**Chr**	**Position**	**Region relative to gene**	**Allele (minor/major)**	**Minor allele frequency (case/control)**	**OR (95% CI)**	***P*-value**
*TMEM158*	rs118109628	3	45279523	Downstream, upstream	A/G	0.04/0.002	20.95 (4.56, 96.32)	3.26E−08
*C21orf37*	rs208892	21	18813490	Intron	A/G	0.43/0.25	2.26 (1.67, 3.05)	6.84E−08
*PCSK6*	rs12908851	15	102042815	Intron, upstream, downstream	T/C	0.07/0.01	6.82 (3.05, 15.24)	8.15E−08
*DUSP26*	rs147270897	8	34132814	Intron, upstream	C/T	0.05/0.01	8.62 (3.20, 23.24)	3.89E−07
*ZNF138*	rs117999072	7	64228326	Upstream, downstream	A/G	0.09/0.02	4.33 (2.32, 8.08)	6.42E−07
*CHD9*	rs149201992	16	52837183	Downstream, upstream	C/T	0.07/0.01	5.81 (2.63, 12.82)	1.04E−06
*HESX1*	rs191751991	3	57241967	Intron, upstream	G/A	0.04/0.003	12.65 (3.40, 47.12)	1.35E−06
*EVA1C*	rs141704048	21	33771938	Upstream, downstream	G/A	0.04/0.003	12.53 (3.36, 46.66)	1.54E−06
*GALNT14*	rs144125291	2	31106055	Downstream, upstream	T/C	0.07/0.01	5.23 (2.48, 11.03)	1.56E−06
*LOC284080*	rs75357358	17	48123894	Downstream, upstream	A/C	0.04/0.003	12.45 (3.34, 46.38)	1.68E−06
*CBFA2T3*	rs150854091	16	89028784	Intron	A/G	0.08/0.02	4.30 (2.24, 8.26)	2.28E−06
*CYP39A1*	rs6907129	6	46597608	Intron	T/G	0.04/0.01	8.36 (2.83, 24.69)	5.10E−06
*CYP39A1*	rs6905960	6	46597262	Intron	G/A	0.04/0.01	8.34 (2.82, 24.64)	5.25E−06
*CYP39A1*	rs7749491	6	46598263	Intron	G/A	0.04/0.01	8.32 (2.82, 24.58)	5.41E−06
*CYP39A1*	rs16874881	6	46596379	Intron	T/A	0.04/0.01	8.30 (2.81, 24.53)	5.57E−06
*RNU6-21P*	rs11648356	16	62206339	Downstream, upstream	T/C	0.07/0.01	4.94 (2.31, 10.53)	5.58E−06
*CA8*	rs72661489	8	60908452	Downstream, upstream	T/C	0.05/0.01	6.32 (2.55, 15.64)	5.85E−06
*MIR378C*	rs60808734	10	132367515	Downstream	C/A	0.05/0.01	6.30 (2.55, 15.61)	6.04E−06
*CDH8*	rs58952871	16	62050053	Intron	C/T	0.07/0.01	4.89 (2.29, 10.43)	6.45E−06
*PLCB1*	rs58120268	20	8120394	Intron	G/A	0.07/0.01	4.89 (2.29, 10.43)	6.45E−06

*SNP, single-nucleotide polymorphism; Chr, chromosome; OR, odds ratio; CI, confidence interval*.

## Discussion

We found several genetic variants that showed associations with motor fluctuations and LID in patients with PD. The occurrence of motor fluctuations was associated with genetic variants in *FAM129B, SNX29, C5orf52*, and *STK10* with *P* < 1.0 × 10^−5^, although the associations were not significant after Bonferroni correction. The occurrence of LID was most significantly associated with *GALNT14* SNP rs144125291, and this association was significant after Bonferroni correction.

The pathophysiology of LID in PD is not well-understood. The functional state of the basal ganglia may be characterized by changes in the neuronal firing rate and oscillatory neuronal activity, which become excessive and possibly have a pathogenic role in the occurrence of abnormal corticostriatal connectivity (16). These mechanisms have been implicated in the pathophysiology of LID in PD. A polymorphism in brain-derived neurotrophic factor, recognized as modulating human cortical plasticity, affects the time to onset of LID in PD in addition to the response to rTMS (17, 18). Further studies using non-invasive brain stimulation techniques may be warranted to clarify the role of those genetic variants in LID.

*GALNT14* SNP rs144125291 is located in the intergenic region 27,276 bases downstream of the gene variant for *GALNT14*. The *GALNT14* gene encodes a Golgi protein that is a member of the polypeptide *N*-acetylgalactosaminyltransferase protein family (19). This enzyme catalyzes the transfer of *N*-acetyl-D-galactosamine to the hydroxyl group on serines and threonines in target peptides (19). Alterations in this gene may play a role in cancer progression and response to chemotherapy in several types of cancer (20–26). Some genes, such as *LRRK2* and *PRKN*, may be associated with both cancer and PD (27–30). *GALNT14* contributes to breast cancer invasion by altering cell proliferation and motility, by altering the expression levels of *EMT* genes, and by stimulating MMP-2 activity (31). MMP-2 is reported to play a role in the inflammatory response (32). *GALNT14* may also cause abundant post-translational modifications, such as glycosylation, which is closely related to tumor growth and metastasis as well as resistance to chemotherapy (33). The development of LID in patients with PD is also related to altered post-synaptic transcription factors and maladaptive plasticity in the nigrostriatal neurons (34). Although the precise pathogenic mechanisms of LID remain unclear, chronic inflammation in the brain and altered post-synaptic plasticity may play key roles in the development of LID (34–36). *GALNT14* SNP rs144125291 may affect the basal level of neuroinflammation in the brain or maladaptive post-synaptic plasticity. However, further functional studies are needed to elucidate the precise role of *GALNT14* in LID.

Several other genes also showed possible association with the occurrence of LID, including *LRPPRC*. *LRPPRC* SNP rs10495912 showed a possible association with LID and is an intergenic variant located 60,028 bases upstream of *LRPPRC*. *LRPPRC* encodes a leucine-rich pentatricopeptide motif-containing protein that predominantly localizes to the mitochondria. The pentatricopeptide repeat (PPR) protein family plays a major role in RNA stability, regulation, processing, splicing, translation, and editing (37). LRPPRC regulates energy metabolism, and the maturation and export of nuclear mRNA. *LRPPRC* mutations have been found to cause Leigh syndrome in a French–Canadian population and are associated with reduced levels of LRPPRC and lower steady-state levels of mitochondrial transcripts (38). Leigh syndrome is an inherited neurometabolic disorder characterized by the occurrence of severe and deadly acidotic crises due to a tissue-specific deficiency in cytochrome c oxidase (38). An *LRPPRC* intronic variant can affect the normal splicing of *LRPPRC* and has been associated with susceptibility to PD (39). Mitochondrial susceptibility in the putamen is reported to play a role in the development of dyskinesia in patients with PD (40), suggesting that abnormal energy metabolism caused by *LRPPRC* variants may be associated with the occurrence of LID. However, further genetic and functional studies are needed to elucidate the role of *LRPPRC* in the development of LID.

Of the genes associated with the occurrence of motor fluctuations, *FAM129B* showed the lowest *P*-value (OR = 2.93, 95% CI = 1.8–4.8, *P* = 6.5 × 10^−6^). Knockdown of FAM129B in HeLa cells accelerates the onset of apoptosis induced by TNF-α (41). Activation of the inflammatory response is closely associated with the pathogenesis of PD, and the increased release of pro-inflammatory cytokines such as TNF-α, interleukin-1β, and interferon-γ has been observed in the post-mortem brain of a PD patient (42). In addition to susceptibility to PD, neuroinflammation in the striatum as well as in the substantia nigra pars compacta may play an important role in the development of motor fluctuations in PD via presynaptic and post-synaptic mechanisms. The storage hypothesis for motor fluctuations posits that the loss of presynaptic dopaminergic terminals reduces the capacity for storage of dopamine in the striatum, thereby inhibiting the ability to compensate for oscillations in plasma levodopa levels, and neuroinflammation may contribute to this effect (43). Neuroinflammation and chronic overproduction and abnormal release of TNF-α by microglia may also contribute to the post-synaptic mechanisms of motor fluctuations, which may be associated with complex striatal functional abnormalities in basal ganglia motor circuits (44). Further functional studies are necessary to investigate the precise role of *FAM129B* in neuroinflammation in PD.

*TBC1D5*, which showed a possible association with the occurrence of motor fluctuations in patients with PD aged over 50 years, functions as a GTPase-activating protein for RAB7 and inhibits recruitment of the VPS35/VPS29/VPS26 subcomplex to membranes (45). The retromer complex is a key component of the endosomal protein sorting machinery and mediates cargo selection through a trimeric complex comprising VPS35/VPS29/VPS26, which is recruited to endosomes by binding to RAB7a and SNX3 (46). This retromer function is closely linked to PD. *VPS35* mutations are a rare cause of autosomal dominant late-onset PD. The clinical features of PD with *VPS35* mutations were as follows: lower onset age, good response to levodopa, and motor complications (47). *VPS13C* mutations are a rare cause of autosomal recessive early-onset PD. The clinical features of PD with *VPS13C* mutations suggested that the progression is rapid and severe (48). Thus, *VPS*-related variants might be associated with motor complications in patients with PD. *RYR2*, which also associates with the occurrence of motor fluctuations in patients with PD aged over 50 years (*P* = 2.5 × 10^−5^), encodes a ryanodine receptor. Ryanodine receptors are intracellular calcium release channels found in the endoplasmic reticulum of all cells, with RYR2 predominating among the three isoforms (RYR1, RYR2, and RYR3) (49). When cellular Ca^2+^-regulating systems are compromised, synaptic dysfunction, impaired plasticity, and neuronal degeneration occur, such as in PD (50). Functional studies are needed to clarify the roles of *TBC1D5* and *RYR2* in the occurrence of motor fluctuations in PD.

The genetic association studies using a small number of pre-specified genetic region were able to determine the genetic risk variants for LID. A previous study reported that the Val158Met variant of catechol-*O*-methyltransferase was associated with LID (51). In another previous study, 229 (45.5%) of 503 Korean patients with PD experienced LID during the mean disease duration of 10.9 years (52). In their candidate gene association study, only the p.S9G variant of dopamine receptor D3 was associated with the occurrence of diphasic dyskinesia (52). However, these studies had limitations as only a limited number of candidate genes were selected due to their incomplete understanding of the pathophysiology of motor complications. Our GWAS investigated a genome-wide set of genetic variants, and this hypothesis-free GWAS may provide a comprehensive evaluation of genetic risk factors for motor complications.

This study has limitations. First, our study used retrospective clinical data. Motor fluctuations and LID are closely related to the pattern and dosage of dopaminergic medications, which were not randomized due to the inherent limitations of a retrospective study. The prevalence of motor fluctuations (29.6%) and LID (23.2%) was slightly lower in the present study than in the previous clinical studies (53, 54); however, this rate of motor complications may be dependent on the patterns of prescribing dopaminergic medications (55, 56). Recently, the prevalence of motor complications is now ~20–28%, which is comparable to what we observed (57, 58). Motor fluctuations and LID are complex phenomena where several factors may contribute to their development and further studies are required to better understand their pathophysiology (59). Second, we assessed the UPDRS for the evaluation of LID, but we did not use more specific assessment tools, such as Unified Dyskinesia Rating Scale, due to practical issues. Hence, future studies should perform a more detailed clinical assessment of LID. Third, our sample size was small compared with that of the traditional GWAS. Deep phenotyping in larger samples is challenging; thus, a well-designed GWAS on clinically important issues should be conducted.

In conclusion, this study provides new insights into the genetic contributions to motor fluctuations and LID in PD. Future collaborative longitudinal genomic studies are needed to further investigate the genetic risk factors associated with motor fluctuations and LID in patients with PD.

## Data Availability Statement

The datasets presented in this study can be found in online repositories: https://www.ncbi.nlm.nih.gov/SNP/snp_viewBatch.cgi?sbid=1063124. The names of the repository/repositories and accession number(s) can be found in the article/Supplementary Material.

## Ethics Statement

The studies involving human participants were reviewed and approved by the Institutional Review Board (IRB) of Asan Medical Center. The patients/participants provided their written informed consent to participate in this study.

## Author Contributions

SC contributed to the conception, organization, execution of the research project, design, execution, review, critique of the statistical analysis, writing of the first draft, and review and critique of the manuscript. H-SR contributed to the execution of the research project, design, execution, review, critique of the statistical analysis, writing of the first draft, and review and critique of the manuscript. KP, NC, JiK, Y-MP, SJ, M-JK, YK, JuK, KK, and S-BK contributed to the execution of the research project, design, execution, review, critique of the statistical analysis, and review and critique of the manuscript.

## Conflict of Interest

The authors declare that the research was conducted in the absence of any commercial or financial relationships that could be construed as a potential conflict of interest.
