# Supplementary material for: Genomic Analysis Identifies New Loci Associated With Motor Complications in Parkinson's Disease
Source: Front Neurol. 2020 Jul 7;11:570. doi: 10.3389/fneur.2020.00570 (PMC7358548; doi:10.3389/fneur.2020.00570)
Supplement: Supplementary file 1 [file Data_Sheet_1.DOCX]

**SNP data access**

DATA UPLOADED TO: NCBI, dbSNP Short Genetic Variations

BATCH TOTAL COUNT: 69

ACCESSION NUMBERS:

ss2137544091
ss3984446715
ss3984446716
ss3984446717
ss3984446718
ss3984446719
ss3984446720
ss3984446721
ss3984446722
ss3984446723
ss3984446724
ss3984446725
ss3984446726
ss3984446727
ss3984446728
ss3984446729
ss3984446730
ss3984446731
ss3984446732
ss3984446733
ss3984446734
ss3984446735
ss3984446736
ss3984446737
ss3984446738
ss3984446739
ss3984446740
ss3984446741
ss3984446742
ss3984446743
ss3984446744
ss3984446745
ss3984446746
ss3984446747
ss3984446748
ss3984446749
ss3984446750
ss3984446751
ss3984446752
ss3984446753
ss3984446754
ss3984446755
ss3984446756
ss3984446757
ss3984446758
ss3984446759
ss3984446760
ss3984446761
ss3984446762
ss3984446763
ss3984446764
ss3984446765
ss3984446766
ss3984446767
ss3984446768
ss3984446769
ss3984446770
ss3984446771
ss3984446772
ss3984446773
ss3984446774
ss3984446775
ss3984446776
ss3984446777
ss3984446778
ss3984446779
ss3984446780
ss3984446781
ss3984446782
