# Supplementary material for: Genomic Analysis Identifies New Loci Associated With Motor Complications in Parkinson's Disease
Source: Front Neurol. 2020 Jul 7;11:570. doi: 10.3389/fneur.2020.00570 (PMC7358548; doi:10.3389/fneur.2020.00570)
Supplement: Supplementary file 2 [file Data_Sheet_2.docx]

Supplementary Material

| **Supplementary Table 1.** Comparison of patients with Parkinson’s disease (PD) with and without motor fluctuations at 5 years after disease onset | | | |
| --- | --- | --- | --- |
|  | PD patients (n = 741) | | |
|  | Motor fluctuations (+) | Motor fluctuations (-) | *P*-value |
| Total sample, n (%) | 219 (29.6) | 522 (70.4) | - |
| Men, n (%) | 92 (42.0) | 233 (44.6) | 0.480 |
| Age at PD onset, years, mean ± SD (range) | 54.0 ± 10.3 (28–82) | 58.4 ± 9.7 (28–87) | <0.001 |
| Disease duration, years, mean ± SD (range) | 9.5 ± 4.0 (5–25) | 11.3 ± 4.6 (5–31) | <0.001 |
| MMSE score, mean ± SD (range) | 26.4 ± 3.3 (12–30) | 25.9 ± 3.2 (10–30) | 0.010 |
| MoCA score, mean ± SD (range) | 23.0 ± 6.1 (3–30) | 22.4 ± 5.3 (3–30) | 0.069 |

PD, Parkinson’s disease; SD, standard deviation; MMSE, Mini–Mental State Examination; MoCA, Montreal Cognitive Assessment.

| **Supplementary Table 2.** Comparison of patients with Parkinson’s disease (PD) with and without levodopa-induced dyskinesia at 5 years after disease onset | | | |
| --- | --- | --- | --- |
|  | PD patients (n = 741) | | |
|  | LID (+) | LID (-) | *P*-value |
| Total sample, n (%) | 172 (23.2) | 569 (76.8) | - |
| Men, n (%) | 75 (43.6) | 250 (43.9) | 0.892 |
| Age at PD onset, years, mean ± SD (range) | 55.2 ± 10.7 (28–79) | 57.7 ± 9.8 (29–87) | 0.007 |
| Disease duration, years, mean ± SD (range) | 9.1 ± 3.5 (5–20) | 11.3 ± 4.6 (5–31) | <0.001 |
| MMSE score, mean ± SD (range) | 26.4 ± 3.0 (12–30) | 26.0 ± 3.3 (10–30) | 0.130 |
| MoCA score, mean ± SD (range) | 23.0 ± 5.4 (5–30) | 22.4 ± 5.6 (3–30) | 0.328 |

PD, Parkinson’s disease; LID, levodopa-induced dyskinesia; SD, standard deviation; MMSE, Mini–Mental State Examination; MoCA, Montreal Cognitive Assessment.

| **Supplementary Table 3.** Comparison of patients aged ≥50 years at onset of Parkinson’s disease (PD), with and without motor fluctuations at 5 years after PD onset | | | | | |
| --- | --- | --- | --- | --- | --- |
|  | PD patients aged ≥50 years at PD onset  (n = 578) | | | | |
|  | | Motor fluctuations (+) | Motor fluctuations (-) | *P*-value |  |
| Total sample, n (%) | | 141 (24.4) | 437 (75.6) | - |  |
| Men, n (%) | | 55 (39.0) | 192 (43.9) | 0.276 |  |
| Age at onset, years, mean ± SD (range) | | 60.0 ± 7.0 (50–82) | 61.5 ± 7.1 (50–87) | 0.024 |  |
| Disease duration, years, mean ± SD (range) | | 9.1 ± 3.5 (5–21) | 10.7 ± 3.9 (5–27) | <0.001 |  |
| MMSE score, mean ± SD (range) | | 28.8 ± 3.7 (12–30) | 25.8 ± 3.1 (10–30) | 0.343 |  |
| MoCA score, mean ± SD (range) | | 21.6 ± 6.7 (3–30) | 21.9 ± 5.4 (3–30) | 0.752 |  |

PD, Parkinson’s disease; SD, standard deviation; MMSE, Mini–Mental State Examination; MoCA, Montreal Cognitive Assessment.

| **Supplementary Table 4.** Comparison of patients aged ≥50 years at onset of Parkinson’s disease (PD), with and without levodopa-induced dyskinesia at 5 years after PD onset | | | |  |
| --- | --- | --- | --- | --- |
|  | PD patients aged ≥50 years at PD onset  (n = 578) | | | |
|  | LID (+) | LID (-) | *P*-value | |
| Total sample, n (%) | 115 (19.9) | 463 (80.1) | - | |
| Men, n (%) | 51 (44.3) | 196 (42.3) | 0.750 | |
| Age at onset, years, mean ± SD (range) | 61.1 ± 7.2 (50–79) | 61.2 ± 7.0 (50–87) | 0.967 | |
| Disease duration, years, mean ± SD (range) | 8.9 ± 3.2 (5–20) | 10.7 ± 3.9 (5–27) | <0.001 | |
| MMSE score, mean ± SD (range) | 26.2 ± 3.1 (12–30) | 25.7 ± 3.3 (10–30) | 0.138 | |
| MoCA score, mean ± SD (range) | 22.0 ± 5.9 (5–30) | 21.8 ± 5.7 (3–30) | 0.687 | |

PD, Parkinson’s disease; LID, levodopa-induced dyskinesia; SD, standard deviation; MMSE, Mini–Mental State Examination; MoCA, Montreal Cognitive Assessment.

| **Supplementary Table 5.** Sex difference in frequency of motor complications at 5 years after onset of Parkinson’s disease | | | | |  |
| --- | --- | --- | --- | --- | --- |
|  | PD patients (n = 741) | | | | |
|  | | Men | Women | *P*-value | |
| Total sample, n (%) | | 325 (43.9) | 416 (56.1) | - | |
| Age at onset, years, mean ± SD (range) | | 56.9 ± 10.6 (28–87) | 57.3 ± 9.7 (29–82) | 0.526 | |
| Disease duration, years, mean ± SD (range) | | 10.7 ± 4.1 (5–26) | 10.9 ± 4.8 (5–31) | 0.598 | |
| Patients with motor fluctuations at 5 years after PD onset, n (%) | | 92 (28.3) | 127 (30.5) | 0.480 | |
| Patients with levodopa-induced dyskinesia at 5 years after PD onset, n (%) | | 75 (23.1) | 97 (23.3) | 0.892 | |

PD, Parkinson’s disease.
