# Supplementary figures and images for: Genomic Analysis Identifies New Loci Associated With Motor Complications in Parkinson's Disease
Source: Front Neurol. 2020 Jul 7;11:570. doi: 10.3389/fneur.2020.00570 (PMC7358548; doi:10.3389/fneur.2020.00570)

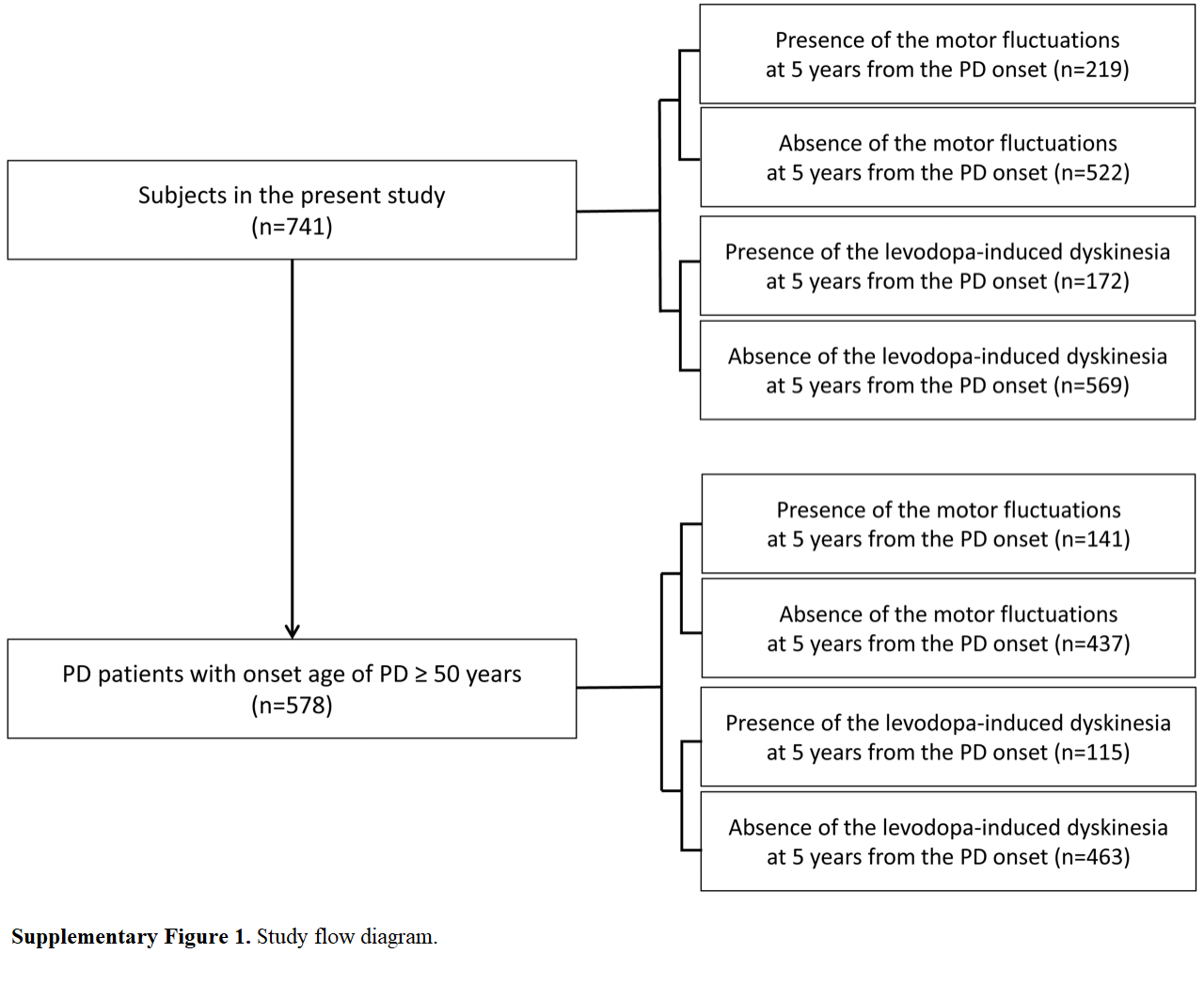

Supplement: Supplementary file 3 [file Image_1.TIF]

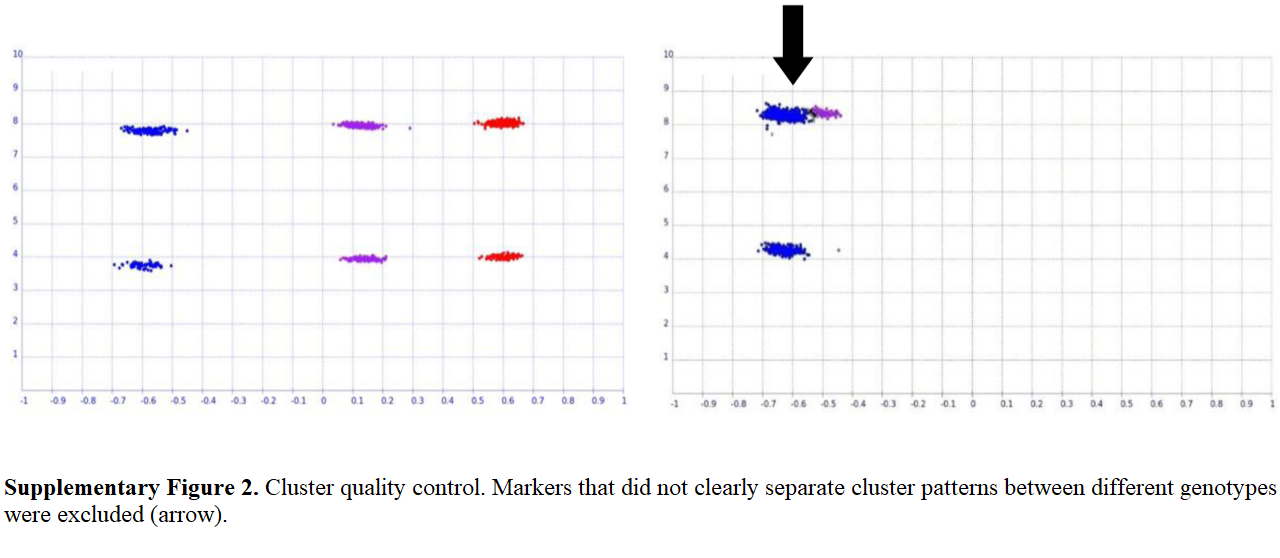

Supplement: Supplementary file 4 [file Image_2.TIF]

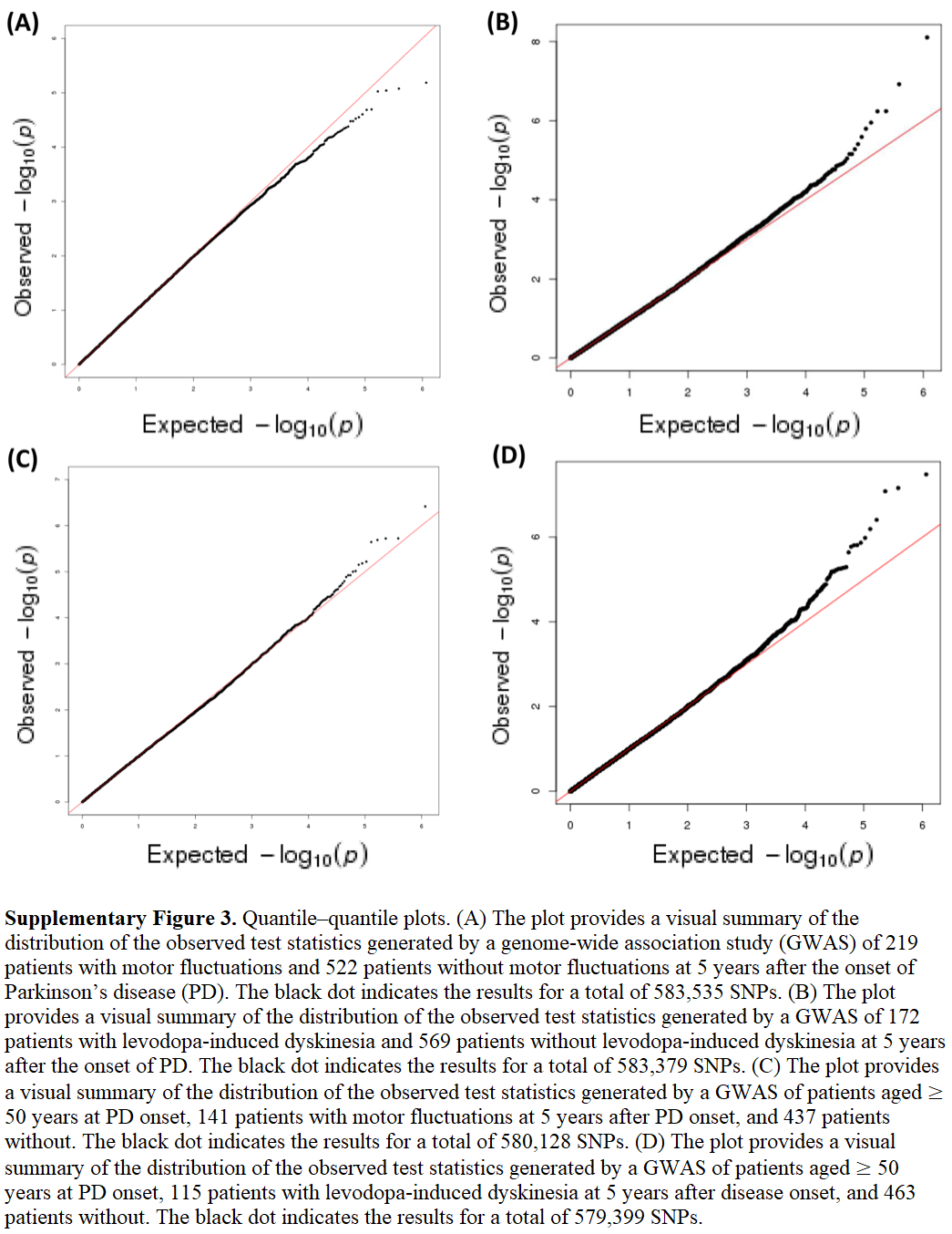

Supplement: Supplementary file 5 [file Image_3.TIF]

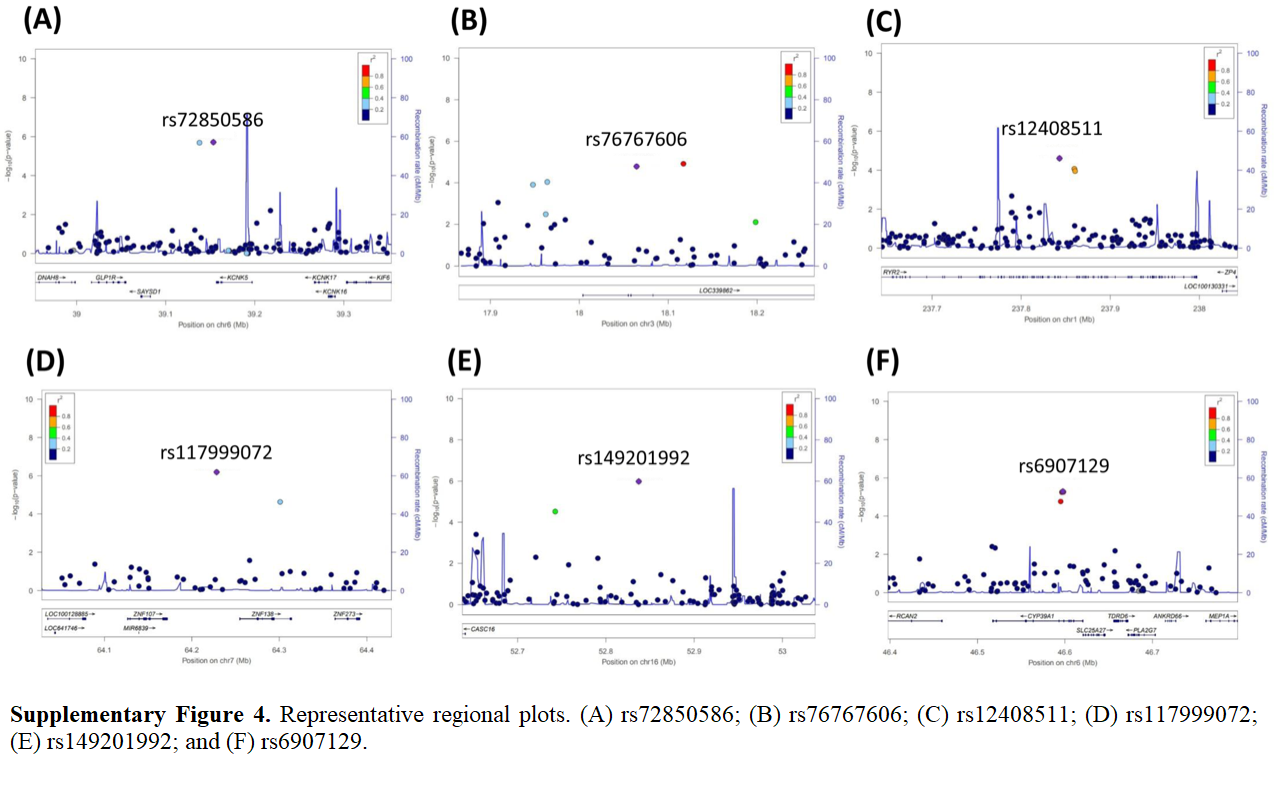

Supplement: Supplementary file 6 [file Image_4.TIF]
